# Effects of Dietary Supplementation with *Caesalpinia sappan* Linn. Extract for Promoting Flock Health and Performance in Late-Phase Laying Hens

**DOI:** 10.3390/ani14030515

**Published:** 2024-02-04

**Authors:** Methisa Longchuphon, Peerawit Chongrattanameteekul, Raktham Mektrirat, Korawan Sringarm, Wanaporn Tapingkae, Orranee Srinual, Kiattisak Huanhong, Wipasiri Chaiphun, Chaiwat Arjin, Sanchai Jaturasitha, Chompunut Lumsangkul

**Affiliations:** 1 Department of Animal and Aquatic Sciences, Faculty of Agriculture, Chiang Mai University, Chiang Mai 50200, Thailand; methisa_longchuphon@cmu.ac.th (M.L.); korawan.s@cmu.ac.th (K.S.); wanaporn.t@cmu.ac.th (W.T.); orranee.s@cmu.ac.th (O.S.); kiattisak_huanhong@cmu.ac.th (K.H.); wipasiri.s@cmu.ac.th (W.C.); chaiwat.arjin@cmu.ac.th (C.A.); 2 Department of Veterinary Biosciences and Public Health, Faculty of Veterinary Medicine, Chiang Mai University, Chiang Mai 50100, Thailand; peerawit_ch@cmu.ac.th; 3 Research Center for Veterinary Biosciences and Veterinary Public Health, Faculty of Veterinary Medicine, Chiang Mai University, Chiang Mai 50100, Thailand; 4 Center of Excellence in Pharmaceutical Nanotechnology, Faculty of Pharmacy, Chiang Mai University, Chiang Mai 50200, Thailand; 5 Research Unit for Innovation in Responsible Food Production for Consumption of the Future (RIFF), Multidisciplinary Research Institute, Chiang Mai University, Chiang Mai 50200, Thailand; 6 Cluster of Agro Bio-Circular-Green Industry, Faculty of Agro-Industry, Chiang Mai University, Chiang Mai 50100, Thailand; 7 Multidisciplinary Research Institute, Chiang Mai University, Chiang Mai 50200, Thailand; sanchai.j@cmu.ac.th

**Keywords:** antioxidant, anti-inflammation, egg quality, feed additive, immunostimulant, laying hen, livestock production, supplement

## Abstract

**Simple Summary:**

The decline in productive performance and egg quality reported in the late stages of the egg-laying cycle is primarily related to oxidative stress and an inflammatory response from prolonged egg production. The present study investigated the effects of dietary supplementation of *Caesalpinia sappan* Linn. Extract (CSE) on flock health and productive performance in late-phase laying hens. The CSE-supplemented groups improved significantly in egg weight, albumin weight, and intestinal structure, as well as a decrease in inflammation and an increase in antioxidant capacity. No adverse effects on blood parameters or organ weights were also observed. These findings suggest that phytogenic CSE has the potential to enhance the health and performance in laying hens, offering valuable insights for the poultry industry. This also is particularly significant given the restrictions on the use of antimicrobial and chemical agents in livestock production.

**Abstract:**

The present study investigated the effects of dietary supplementation of *Caesalpinia sappan* Linn Extract (CSE) on the health and productive performance of late-phase laying hens on farms. Proximate composition and antioxidant markers of CSE powder revealed favorable characteristics with high total dry matter; phenolic content, and antioxidant potency. Three hundred and sixty (64-week-old) Hy-line Brown hens were divided into five groups with 0 (control diet), 250, 500, 1000, and 2000 mg/kg CSE, respectively. The laying performance and egg quality of the CSE supplementation groups demonstrated significant improvements in egg weight and albumin weight (*p* < 0.05), and a tendency for enhanced egg mass and feed conversion ratio. Additionally, the intestinal morphostructural indices in the 2000 mg CSE/kg diet group showed the greatest statistical significance (*p* < 0.05), with a detectable trend suggesting an increase in the villus height to crypt depth ratio. In addition, significant downregulation of proinflammatory genes occurred in their liver tissues, coupled with a greater expression of genes linked to antioxidants and anti-inflammatory processes. Furthermore, the blood biochemical parameters and the organ weights may suggest a favorable safety profile of CSE supplementation. These findings highlight the potential of CSE as a dietary supplement to enhance the productive performance and flock health of late-phase laying hens. Further research is warranted to explore the long-term effects and optimal dosage of CSE supplementation for laying hens in farming practices

## 1. Introduction

For high-protein food, egg products have become common as economically viable options for the nutritional needs of humans throughout the world. Commercial poultry production has undergone significant industrialization, resulting in extended feeding periods. As a result, the physiological processes of laying hens progressively reduce following their peak laying phase, leading to a rapid decline in their productive performance and egg quality during the late laying period [1]. The decreased yield and quality is predominantly due to the accumulation of inflammatory and oxidative stress during a long-term production of eggs [2]. Moreover, the limitations on antimicrobial feed additives for laying hens have posed both challenges and opportunities for the poultry industry [3]. The efficient utilization of non-natural feed sources is also constrained, and these issues are frequently associated with consumer apprehensions. In addition, this limitation requests an innovation of ecologically appropriate, organic, and effective alternatives to improve animal health and performance [4,5].

Nutraceuticals and phytogenic feed additives, which are naturally occurring chemical compounds, have been demonstrated to improve the physiological and productive features in poultry [6,7,8,9]. Remarkably, Sappan wood (*Caesalpinia sappan* L.) has been previously documented to possess antibacterial properties when used as a feed additive, impacting the intestinal microflora of laying quail. Additionally, *C. sappan* belongs to the Leguminosae family and is a tropical tree native in tropical Asian regions such as Southern China, India, Sri Lanka, Myanmar, Vietnam, and Thailand. *C. sappan* contains a water-soluble compound called brazilin, which is used in the production of food and beverages [10]. The dried heartwood of *C. sappan* is also traditionally used in ethnopharmacology such as Chinese, Indian, and Thai Traditional remedies. Interestingly, *C. sappan* has been reported to possess various biological activities including antioxidative [11,12], anti-inflammatory [13,14], immunomodulatory [15], anti-allergic [16], analgesic [17], antibacterial [18], and antiviral activities [19].

The hypothesis is based on the reported antioxidative and immunomodulatory activities of *C. sappan*, which suggest its potential to mitigate the accumulation of inflammatory and oxidative stress in laying hens during their late laying period. In addition, the present study aimed to investigate the potential of using phytogenic ingredients from CSE to alleviate inflammatory and oxidative stress, which can impact the health and productivity of late-phase laying hens. The assessment was based on laying performance, egg quality, blood biochemistry parameters, indices of morphostructure intestinal villi, and the expressions of immune and antioxidant-related genes.

## 2. Materials and Methods

### 2.1. Animals and Ethical Approval

A total of 360 64-week-old Hy-line Brown hens were obtained from the stock of a commercial layer farm (8 birds/m^2^), Chiang Mai, Thailand. The layers were housed with strict biosecurity measures in Evaporative Emission Control System with 24 ± 1.0 °C, 65 ± 2.0% relative humidity, and 16 h controlled daily light. Four hens were kept in an individual cage (41 × 46 × 40 cm) in the second of 3-tier ladder-type cages. Throughout the 8 weeks of the experiment, the laying hens were equipped with one feeder and two nipple drinkers ad libitum. All the experimental procedures in this study were conducted in strict accordance with the guidelines recommended and ethically approved by the Animal Ethics Committee, Faculty of Agriculture, Chiang Mai University with the protocol number RAGIACUC011/2565.

### 2.2. Plant Material

Powdered extract of CSE was provided from the Specialty Natural Products Co., Ltd. (Chonburi, Thailand). The Integrated Taxonomic Information System (ITIS) report provides the taxonomic serial number 506349, while the International Plant Names Index (IPNI) presents nomenclatural data associated with plant ID 482900-1.

### 2.3. Antioxidant Analysis

The CSE was analyzed for total phenolic content (TPC) using the Folin–Ciocalteu procedure [20]. The extract was mixed with Folin–Ciocalteu reagent and 7.5% (*w*/*v*) NaCO_3_ solution. After incubating for 60 min, the calibration standard for gallic acid was established using a UV-Vis spectrophotometer (SPECTROstar Nano, BMG LABTECH, Ortenberg, Germany). The total phenolic content of the extract was then quantified in milligrams of gallic acid per gram. The antioxidant activities of CSE were also evaluated by spectrophotometric ferric-reducing antioxidant power (FRAP), 2,2-diphenyl-1-picrylhydrazyl (DPPH), 2,2′-azino-bis (3-ethylbenzothiazoline-6-sulfonic acid) (ABTS) radical scavenging methods reported with some modifications [21]. A series of Trolox solutions with varying concentrations (ranging from 0 to 15 mM) were prepared using 80% methanol, and the absorbance of each solution was measured to create a standard curve. The absorbance measurements were conducted with a microplate reader Synergy H1 (BioTek, Winooski, VT, USA).

### 2.4. Proximate Composition Analysis

The basal diet and CSE were analyzed to assess their proximate composition. The analytical determinations were conducted in triplicate. The dry matter content (DM) of the diet samples was measured using gravimetry, specifically the AOAC technique n. 934.01. The ash content (ASH) was determined using gravimetric analysis following sample incineration using a muffle furnace, according to the AOAC method n. 942.05. The Kjeldahl method (AOAC method n. 978.04) was used to analyze the crude protein (CP), while the ether extract (EE) was determined using Soxhlet extraction (AOAC method n. 920.39). The acid detergent fiber was measured according to the method outlined by Goering and Van Soest [22]. The gross energy (MJ/kg DM) of the food samples was determined using a Parr 6200 Isoperibol Calorimeter (Parr Instrument Company, Moline, IL, USA) following the instructions provided by the manufacturer.

### 2.5. Dietary Preparation and Feeding

A basal diet consisted in a commercial standard layer diet for hens in late phase of laying cycle. The composition and nutrient content of the basal diet, which has a gross energy of 3105.33 kcal/kg, are detailed in Table 1. The CSE powder was evenly sprinkled over a small portion of basal feed and thoroughly mixed. Subsequently, this mixture was carefully integrated into the required amount of feed, ensuring complete homogenization by feed mixer machine (Model MI1HP3V01, Siam Farm Service Co, Ltd., Lampang, Thailand) to achieve the final concentrations of CSE at 250, 500, 1000, and 2000 mg/kg of diet. The laying hens were randomly assigned into 5 groups, with 6 replicates per treatment and 12 hens per replicate. The control group received a standard basal diet, while the 4 treatment groups received the different diets supplemented with CSE.

The basal diet consisted in a commercial pelleted diet for layer. The composition of the basal diet used in this study is shown in Table 1.

### 2.6. Productive Performance and Egg Quality

Individual hen bodyweight data were recorded at the beginning and end of the trial. Daily measurements of the feed provided and residual feed were taken to analyze feed intake (FI) and average daily feed intake (ADFI). The number of eggs and egg weight were recorded every day to measure egg production efficiency and the feed convention (FCR) was calculated as feed consumption divided by the total egg mass (feed/egg mass, g/g). Records of the overall health and mortality of laying hens were kept over the course of the experiment. At week 1 to 8, 5 eggs/replicate were collected each week for analyzing the egg quality. Eggshell thickness was measured by using a digital vernier caliper. Eggshell, albumin, and yolk weight were measured by using a digital scale. To determine another egg quality, the Digital Egg Tester DET 6000 (NABEL Co., Ltd., Kyoto, Japan) was utilized to evaluate the egg weight, eggshell strength, albumen height, Haugh unit (HU), and yolk color.

### 2.7. Serum Biochemical Analysis

At the end of experiment, three laying hens per replicate were randomly selected for blood collection. Birds are euthanized via carbon dioxide. In total, 3 mL of blood sample was drawn from the brachial wing using a heparin sodium anticoagulant tube. For biochemical analysis, including alanine aminotransferase (ALT), alkaline phosphate (ALP), aspartate aminotransferase (AST), total bilirubin (TBIL), direct bilirubin (DBIL), indirect bilirubin (IBIL), total protein (TP), albumin (ALB), and globulin (GLB), a Sysmex BX-3010 automated analyzer from Kobe, Japan, was used to measure blood biochemical parameters. The serum was isolated by subjecting the samples to centrifugation at a speed of 2200× *g* for a duration of 15 min using a Kokusan H-19α centrifuge located in Kokusan, Saitama, Japan. Prior to centrifugation, the serum was allowed to stabilize at room temperature for 30 min.

### 2.8. Pathological Evaluation and Intestinal Histomorphometry

At the end of the feeding trial, necropsies were performed on three laying hens per replicate. Their vital organs and portions of three small intestine segments, including the duodenum, jejunum, and ileum, were collected. The organ weight of each chicken was recorded. The tissues were initially preserved with 10% neutral-buffered formalin, and then a series of processes was performed which consisted of dehydration, clearing, and impregnation. The tissues fixed in paraffin were sliced into 4.5 µm sections using a rotary microtome. The specimens were then treated to remove the paraffin and stained with hematoxylin and eosin (H&E). The compound microscope (CX21, Olympus Corporation, Tokyo, Japan) with a digital video camera (Motic MC 2000) was used to visualize the length of morphometric villi and the depth of crypts. The digital photographs of intestinal tissues were subsequently examined using an image analyzer to quantify the dimensions of villus height (VH; μm), villus width (VW; μm), crypt depth (CD; μm), and crypt area (CA; μm^2^).

### 2.9. Analysis of Immune and Antioxidant-Related Gene Expressions

The liver tissues were randomly selected from three laying hens per replicate. The liver tissues were immediately removed and frozen at −20 °C until RNA extraction. Fifty milligrams of samples were mixed with lysis buffer and homogenized using a tissue homogenizer. RNA extraction was then carried out according to manufacturer’s protocol using a column-based RNA extraction kit (Invitrogen, PureLinkTM RNA Mini Kit, MA USA). The concentration and purity of total RNA was measured at an absorbance ratio of 260–280 nm using NanoDrop2000 spectrophotometer (Thermo Fisher Scientific, Waltham, MA, USA). The cDNA was synthesized using Bio-Rad iScriptTM RT Supermix cDNA synthesis kit (Bio-Rad, Hercules, CA, USA). The primers required for *IL-1β*, *IL-6*, *IL-10*, *TNF*-α, *SOD*, *CAT*, *GSH-Px1*, and *Nrf2* genes were designed according to Table 2. Employing the CFX Connect^TM^ Real-Time PCR System (Bio-Rad, USA) and the iTaq Universal SYBR Green supermix 2× (Bio-Rad, USA) in addition to the specific primers for individual genes, the qPCR reaction was conducted. The expression levels of the antioxidant and immune-related gene were measured using the 2^−ΔΔCt^ method and a standard curve [23].

### 2.10. Statistical Analysis

The data were described using descriptive statistics, which comprised measures such as frequency, proportion, and mean with standard deviation. The normality assumption was examined using the Shapiro–Wilk test and visual assessment of the continuous variable distribution using Quantile-Quantile (Q-Q) plots of model residuals. The data were subjected to analysis of variance (ANOVA), followed by the comparison of Least Square Means (LSM) by using Tukey’s test or Kruskal–Wallis H test. Statistical significance was attributed to differences between variables when the bi-caudal probability was less than 5% (*p* < 0.05) attributable to random variation (error type I). The R statistics package was utilized (RStudio, Boston, MA, USA). For graph generation, the GraphPad Prism software version 8.0 (San Diego, CA, USA) was utilized. 

## 3. Results

### 3.1. Proximate Composition and Antioxidant Markers of CSE

The proximate composition of the CSE powder is outlined in Table 3, showing that it consisted of 96.95% total dry matter and 3.05% moisture, along with fiber (27.94%), protein (0.45%), ash (0.32%), and fat (0.04%), respectively. The total phenolic content of CSE was 25.12 mg of gallic acid equivalents per gram (mg GAE/g). Additionally, it exhibited a hydrogen-donating potency of 242.71 μmol Trolox equivalents per gram (μmol TE/g), the capability of single-electron transfer at 7.89 mg/mL, and the ability to reduce ferric ions to ferrous ions at a concentration of 1.75 mM Fe^2+^/g.

### 3.2. Laying Performance

**Figure 1:**
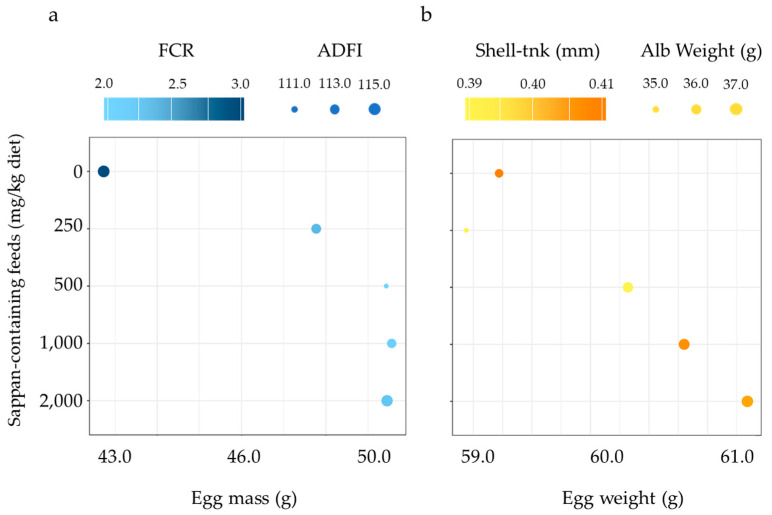
Bubble diagram of functional enrichment analysis for productive performance (**a**) and egg quality (**b**) in late phase of laying cycle 1–8 weeks after diet supplementation by *Caesalpinia sappan* extract with a series of doses in the range 0–2000 mg/kg diet.

Efficacy of CSE on laying performance is shown in Table 4. Significant differences among groups with heavier egg weight were recorded for the group supplemented with 2000 mg CSE/kg diet as compared to the basal diet group during 1–8 weeks of age (*p* = 0.02). Furthermore, there was a tendency for increased egg production, egg number, and egg mass, along with a tendency for the feed conversion ratio to decrease in the four supplementation groups (Figure 1a). Nonetheless, no significant differences were observed among the groups in terms of other egg production parameters over the entire experimental period (*p* > 0.05).

### 3.3. Egg Quality

Efficacy of CSE on the egg quality is shown in Table 5. Supplementation of CSE did not affect yolk weight and eggshell weight (*p* > 0.05). Significant differences among groups with heavier albumin weight were recorded for the groups supplemented with 1000 and 2000 mg CSE/kg diet as compared to the basal diet group during 1–8 weeks of age (*p* < 0.01). These results suggest that the increase in egg weight may be attributed to an increase in albumin weight (Figure 1b). However, the albumin height and Haugh unit were unaffected by CSE supplementation. The shell thickness in the CSE-supplemented groups was significantly lower than that of the basal diet group (*p* < 0.01). Conversely, there was an observed tendency for shell strength to increase in the four supplementation groups.

### 3.4. Blood Biochemical Profiles

For serum protein analysis, significant differences between groups with albumin form the groups supplemented with a 500 mg CSE/kg diet as compared to a 1000 mg CSE/kg diet (*p* = 0.0013). In addition, no significant differences were observed among the basal diet group and four CSE-supplemented groups for the albumin parameter (*p* > 0.05). Moreover, supplementation of CSE did not affect total protein, globulin, all bilirubin parameters, and A/G ratio (*p* > 0.05). For analysis of serum emzymes, no significant effects on Aspartate transaminase (AST), Alanine transaminase (ALT), and Alkaline phosphatase (ALP) were also noted among all treatments (*p* > 0.05) (Table 6).

### 3.5. Organ Weights and Intestine Morphostructure Indices

**Figure 2:**
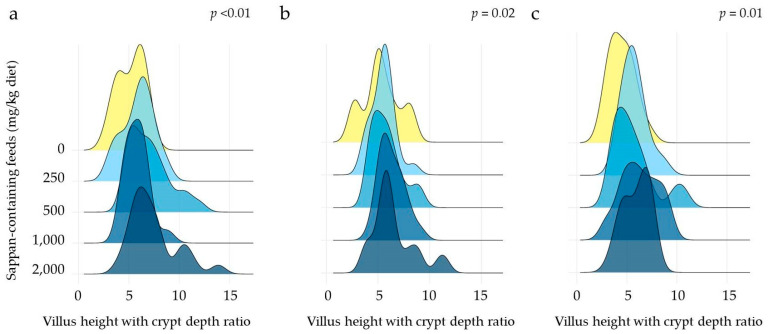
Ridgeline plot of the villus height with crypt depth ratio in different intestinal segments of the duodenum (**a**), jejunum (**b**), and ileum (**c**) from late-phase laying hens at 8 weeks following diet supplementation with *Caesalpinia sappan* extract ranging from 0 to 2000 mg/kg diet. The *p*-values were determined by analyzing the Kruskal–Wallis H test among the groups in each of the different intestinal segments.

No instances of mortality were recorded throughout the duration of this study. After undergoing various dietary regimens, there were no statistically significant differences in the body weight of the laying hens. Similarly, the organ weights of the liver, spleen, heart, gizzard, proventriculus, and intestine also showed no significant variations among the different dietary CSE supplementations (*p* > 0.05) (Table 7). The villus height (VH), villus width (VW), crypt depth (CD), and crypt area (CA) are presented in Table 8 to evaluate the effects of CPE on small intestinal morphological traits. This study found that only the VW of the jejunum and ileum in the group supplemented with the low dose of 250 mg CSE/kg diet were wider than that of the basal diet group. With both medium doses of CSE (500–1000 mg/kg diet), significant differences in more morphostructure indices were observed. Interestingly, all morphostructure indices for duodenum and jejunum showed greater significance in the group supplemented with 2000 mg CSE/kg diet compared to the basal diet group (*p* < 0.05), whereas the mere VW of ileum in the group supplemented with 2000 mg CSE/kg diet was wider than that of the basal diet group (*p* < 0.05). Additionally, there was a noticeable trend of an increase in the villus height to crypt depth ratio (VH:CD) across the three segments of the small intestine in the four supplementation groups (*p* < 0.05) (Figure 2).

### 3.6. Expressions of Immune and Antioxidant-Related Genes

**Figure 3:**
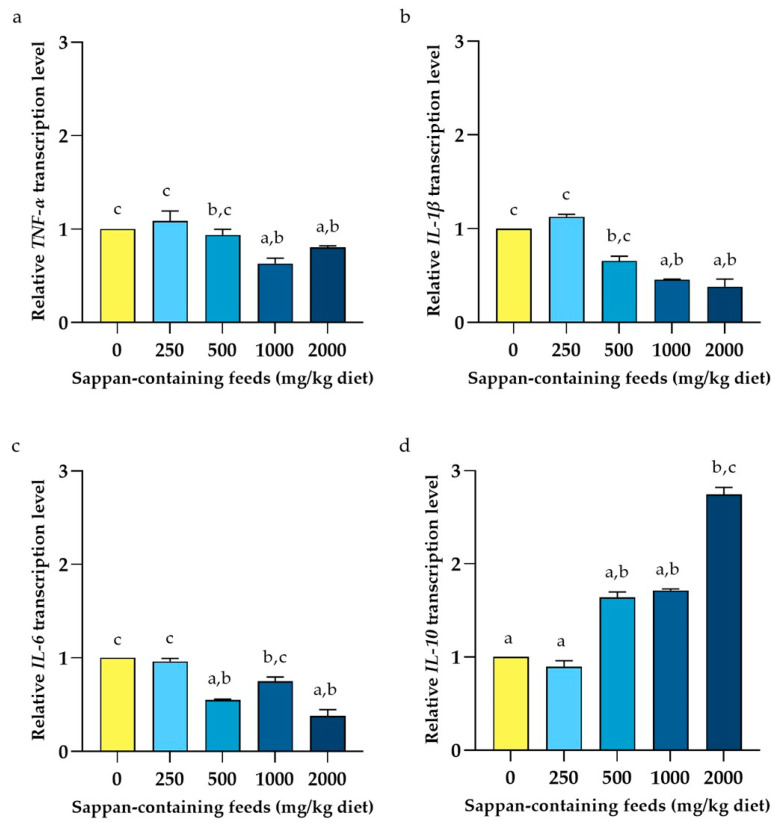
Relative transcript levels (means ± S.D.) of immune-related genes for interleukin-1beta (**a**), interleukin-6 (**b**), tumor necrosis factor-alpha (**c**), and interleukin-10 (**d**) from liver tissue of late-phase laying hens at 8 weeks following diet supplementation with *Caesalpinia sappan* extract ranging from 0 to 2000 mg/kg diet. Data were analyzed by Kruskal–Wallis H test. Bars with different letters indicated values significantly different among the supplemented groups (*p* < 0.05).

**Figure 4:**
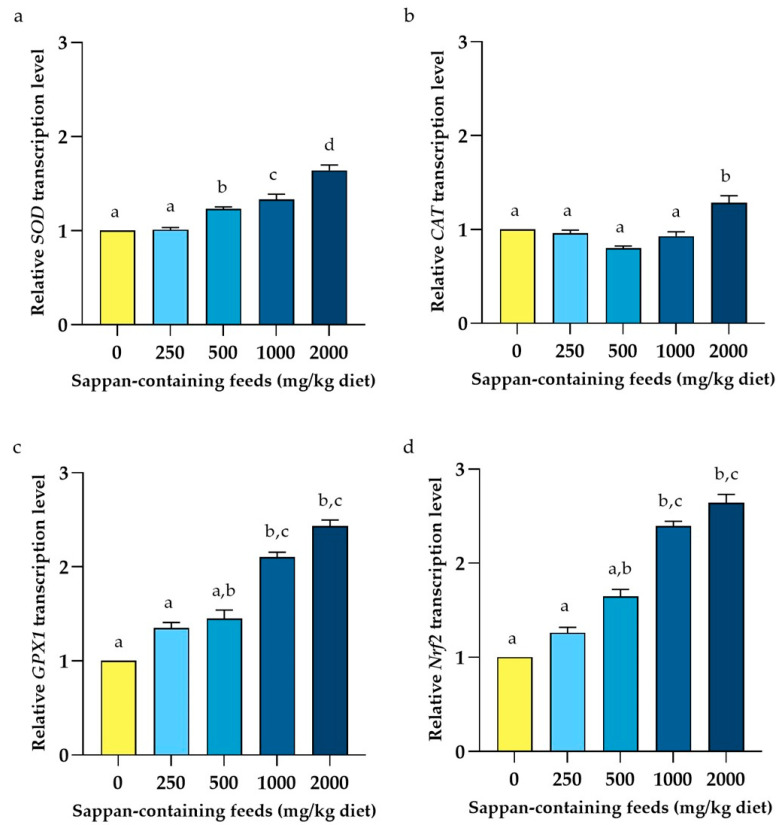
Relative transcript levels (means ± S.D.) of antioxidant-related genes for catalase (**a**), superoxide dismutase (**b**), glutathione peroxidase 1 (**c**), and nuclear factor erythroid 2-related factor 2 (**d**) from liver tissue of late-phase laying hens at 8 weeks following diet supplementation with *Caesalpinia sappan* extract ranging from 0 to 2000 mg/kg diet. Data were analyzed by using Kruskal–Wallis H test. Bars with different letters indicated values significantly different among the supplemented groups (*p* < 0.05).

The effects of CSE on the relative expression of genes involved in the immunity in the liver of laying hens are presented in Figure 3. The results demonstrated that all relative immune-related gene expressions in the group supplemented with 250 mg CSE/kg diet showed no significant differences with the basal diet group (*p* > 0.05). In contrast, the group supplemented with 2000 mg CSE/kg diet displayed significant differences in relative immune-related gene expressions compared to the basal diet group (*p* < 0.05). No significant differences were also observed among the 500, 1000, and 2000 mg CSE/kg diet groups. For the relative expression of antioxidant-related genes, the effects of CSE supplemented in laying hens are illustrated in Figure 4. The results demonstrated that the relative transcript levels of all antioxidant-related genes were observed to increase in a dose-dependent manner. Futhermore, the relative transcription levels of all antioxidant-related genes in the group supplemented with 2000 mg CSE/kg diet were also significantly upregulated compared to the basal diet group (*p* < 0.05).

## 4. Discussion

To ensure the quality of CSE as a feed additive for lying hens, the proximate composition and antioxidant markers of dried CSE powder should be measured. The phytochemical composition of sappan wood has been generally identified, establishing a predominant presence of flavonoids, phenolic acids, and anthraquinones, among additional bioactive compounds [24]. Additionally, the CSE contains a variety of phenolic compounds, such as chlorogenic acid, caffeic acid, and gallic acid. In this study, the total phenolic content of the CSE from Thailand, measured in gallic acid equivalents per gram, aligns with the findings of previous research conducted in Indonesia, which reported a value of 27.65 mg GAE/g [25]. The antioxidant properties of CSE, as determined by FRAP, DPPH, and ABTS, have been demonstrated to be compared with previously published research (FRAP, 78.7 ± 2.4 mM Fe^2+^/mg; DPPH, 51.2 ± 3.2 µM and ABTS, 64.8 ± 4.2 µM/mg) [26,27,28]. The phytochemicals and antioxidant capacity of CSE are determined by the plant ontogenesis; therefore, the quality and quantity of these compounds are influenced by exogenous factors such as the growing season and the timing of harvesting [29]. Furthermore, the amount and phytochemical constitution of CSE are greatly altered by the use of different techniques for extraction and solvents [30,31,32,33].

The reduction in productive performance and egg quantity from laying hens during the late laying stage is affected by the health status acquired according to a consequence of long-term egg production [34]. Despite the lack of significant improvements in overall egg quality parameters, the CSE-supplemented groups exhibited notable enhancements in egg weight, albumin weight, and shell thickness compared to the group on the basal diet. These findings suggest that the CSE supplementation may have specific benefits for certain aspects of egg quality, warranting further investigation into its potential impact on poultry production. These findings are supported by a previous investigation on CSE supplementation in broilers, which demonstrated a positive impact on the growth performance [35]. The administration of CSE could also maintain the body weight for a buck under heat stress [36].

For absorption of dietary components, the microstructure intestinal villi are required to increase the surface area of the intestinal wall, which facilitates better nutrition delivery and efficient digestion enzyme activity [37]. In the present study, dietary CSE supplementation with 2000 mg CSE/kg impacts the intestinal morphology of the duodenum and jejunum in laying hens, including VH, VW, CD, and CA. However, dietary supplementation with 2000 mg CSE/kg increased the VW of the ileum in laying hens compared to that of the basal diet group (*p* < 0.05). Furthermore, a discernible trend of an increase in the ratio of VH:CD was observed across the three segments of the small intestine in the supplemented groups. These parameters are commonly recognized to be vital indicators of the small intestine’s absorption capacity in animals [38,39]. The marked improvements in egg weight and albumin weight observed in the CSE-supplemented groups might be due to the consequences of dietary component absorption, where amino acids are predominantly absorbed in the jejunum [40,41].

After the peak-laying period, the aging process in hens naturally leads to an increase in the production of free radicals and a decrease in scavenging ability [42]. Additionally, insufficient endogenous antioxidants for neutralizing excessive free radicals in the chicken body could disrupt the redox equilibrium, resulting in oxidative stress [43], which may potentially affect egg quality and laying performance [2]. Interestingly, the supplementation of CSE in laying hens exhibited dose-dependent effects for the expression of antioxidant-related genes including *SOD*, *CAT*, *GSH-Px1*, and *Nrf2*. Additionally, nuclear factor erythroid 2-related factor 2 plays a crucial role for initiating the transcription of antioxidant genes. The superoxide dismutase converts superoxide radicals into hydrogen peroxide, which eventually turns into water by catalase, or into non-toxic compounds by glutathione peroxidase [44]. These results agreed with a previous report that the supplementation of CSE increased superoxide dismutase activity and suppressed malondialdehyde levels in rats exposed to inhaled formaldehyde [45]. Furthermore, effects of phenolic substances derived from other plant extracts supplemented to chicken diets on superoxide dismutase activity have been previously described [46].

Alterations in metabolic and hormonal state in late-phase laying hens may contribute to an increased risk of inflammation. For immune-related gene expressions, the group supplemented with 2000 mg CSE/kg diet led to a significant upregulation of anti-inflammatory *IL-10* genes, and a downregulation of pro-inflammatory-related genes including *IL-1β*, *IL-6*, and *TNF*-α. These research findings are consistent with several previous reports that have highlighted the anti-inflammatory properties of *C. sappan* L. in cell-based assays and animal models [47,48,49]. Brazilin, an active compound in CSE, has been demonstrated to downregulate the expression of IRAK4 protein and subsequently leads to inactivate nuclear transcription κB (NF-κB); therefore, the expression of targeted pro-inflammatory cytokines is reduced [50]. Brazilin has an obvious regulatory effect on programmed cell death protein 1 (PD-1)-related IL-10 secretion from monocytes [51].

On the contrary, the safety of CSE was confirmed by the absence of any impact on the blood chemistry parameters of laying hens for the 8-week feeding trial, since the lack of significant effects on AST, ALT, and ALP among all treatments provides valuable insights into the absence of adverse effects on liver function and overall health [52]. Additionally, the maintenance of total protein, globulin, and bilirubin parameters indicate that the CSE supplementation did not induce alterations in liver metabolism and homeostasis [53]. The safety of CSE was further supported by the absence of detectable differences in body weight and organ weights. Additionally, no mortality or signs of intoxication were observed following exposure to various dietary regimens. Furthermore, no chronic, subacute, or acute toxicity of *C. sappan* have been extensively reported [54,55,56].

While the findings of this study suggest positive effects of CSE supplementation on the health and performance of laying hens, it is important to consider some limitations. In addition, the controlled experimental conditions used in this study highlight the important role for future field experiments to examine the practical application and effectiveness of CSE supplementation in a commercial laying hen operation. For potential future research directions, long-term field trials should be conducted to determine the cumulative effect of CSE supplementation. Furthermore, expanding the research scope by addressing the gut flora, as well as investigating CSE bioavailability and pharmacokinetics in laying hens, would be important accomplishments.

## 5. Conclusions

The dietary supplementation of CSE in late-phase laying hens led to an improvement in productive performance, particularly in the parameter of egg weight. Moreover, the supplementation of CSE did not have adverse effects on the serum biochemical parameters and weight of vital organs. The liver tissues of lying hens supplemented with CSE also exhibited a significant upregulation of anti-inflammatory and antioxidant-related genes, coupled with a downregulation of proinflammatory-related genes. The collective findings affirm the immunomodulatory and antioxidative potential of a 2000 mg CSE/kg diet as a beneficial dietary supplement for late-phase laying hens under controlled experimental conditions.

## Figures and Tables

**Table 1:** Basal diet ingredients and chemical composition on a dry matter basis.

Items	Concentration (%)
Ingredients	
Corn	63.6
Soybean meal (44% of CP)	25.6
Calcium carbonate	8.0
Dicalcium phosphate	1.0
DL-methionine	0.5
Sodium chloride	0.3
Premix ^1^	1
Analyzed composition	
Crude protein	16.58
D-Lysine	0.83
Methionine	0.36
Calcium	3.17
Available phosphorus	0.36
Metabolizable enegy (kcal/kg)	3105.33

^1^ Supplied vitamin–mineral premix contains the following per kg diet: vitamin A (12,000 IU), vitamin D3 (2400 IU), vitamin E (30 g), vitamin K3 (2.5 g), vitamin B1 (2.5 g), vitamin B2 (6 g), vitamin B6 (4 g), vitamin B12 (20 mg), niacin (25 g), calcium D-pantothenate (8 g), folic acid (1 g), vitamin C (50 g), D-biotin (50 mg), choline chloride (150 g), canthaxanthin (1.5 g), apo-carotenoic acid ester (0.5 g), manganese (80 g), zinc (60 g), iron (60 g), copper (5 g), iodine (1 g), cobalt (0.5 g), and selenium (0.15 g).

**Table 2:** Primer sequences, amplicons and the related information for quantitative real-time PCR.

Gene	Primer Sequences	Accession No.	Product Size (bp)
*β-actin*	F: CTGGCACCTAGCACAATGAA	X00182.1	109
	R: ACATCTGCTGGAAGGTGGAC		
*IL-1β*	F: TGGGCATCAAGGGCTACA	Y15006	244
	R: TCGGGTTGGTTGGTGATG		
*IL-6*	F: CAAGGTGACGGAGGAGGAC	AJ309540	254
	R: TGGCGAGGAGGGATTTCT		
*IL-10*	F: AGCAGATCAAGGAGACGTTC	NM_001004414.4	103
	R: ATCAGCAGGTACTCCTCGAT		
*TNF-α*	F: TGTGTATGTGCAGCAACCCGTAGT	NM204267	229
	R: GGCATTGCAATTTGGACAGAAGT		
*SOD*	F: CACTCTTCCTGACCTGCCTTACG	NM204211	146
	R: TTGCCAGCGCCTCTTTGTATT		
*CAT*	F: CTGTTGCTGGAGAATCTGGGTC	NM001031215	160
	R: TGGCTATGGATGAAGGATGGAA		
*GPX1*	F: GCGACTTCCTGCAGCTCAACGA	GQ502186.2	99
	R: CGTTCTCCTGGTGCCCGAAT		
*Nrf2*	F: GGAAGAAGGTGCTTTTCGGAGC	NM_205117.1	116
	R: GGGCAAGGCAGATCTCTTCCAA		

*IL*, interleukin; *TNF-α*, tumor necrosis factor alpha; *SOD*, superoxide dismutase; *CAT*, catalase; *GSH-Px1*, glutathione peroxidase 1; *Nrf2*, Nuclear factor erythroid 2-related factor 2.

**Table 3:** Proximate composition and antioxidant activity of Sappan wood extract.

Items	Amounts
Dry matter (%)	96.95
Moisture (%)	3.05
Ash (%)	0.32
Crude protein (%)	0.45
Crude fiber (%)	27.94
Fat (%)	0.04
Total phenolic (mg GE/g)	25.12
DPPH (μmol TE/g)	242.71
ABTS (IC_50_, mg/mL)	7.89
FRAP (mM Fe^2+^/g)	1.75

**Table 4:** The effect of *Caesalpinia sappan* extract supplementation on productive performance of laying hens in late phase of laying cycle 1–8 weeks of experiment.

Variables	Sappan-Containing Feeds (mg/kg Diet)					Pooled SEM	*p*-Value
	0	250	500	1000	2000		
Initial BW (g)	2078.9	2024.7	2102.5	2058.3	2039.0	23.5	0.17
Final BW (g)	2042.0	2031.7	2077.6	2075.8	2014.3	21.7	0.19
ADFI (g)	114.3	112.8	111.02	112.48	114.03	0.62	0.50
FCR	2.72	2.32	2.23	2.25	2.28	0.06	0.08
Egg production (%)	74.23	82.86	83.63	84.85	82.74	1.53	0.19
Egg weight (g)	58.83 ^a^	58.87 ^a^	60.39 ^ab^	59.55 ^ab^	60.99 ^b^	0.26	0.02
Egg mass (g)	43.73	48.77	50.43	50.56	50.45	0.94	0.10

^a,b^ Mean values with different letters in the same row indicate significant differences. SEM, standard error of measurement; ADFI, average daily feed intake; FCR, feed conversion ratio.

**Table 5:** The effect of *Caesalpinia sappan* extract supplementation on egg quality of laying hens in late phase of laying cycle 1–8 weeks of experiment.

Variables	Sappan-Containing Feeds (mg/kg Diet)					Pooled SEM	*p*-Value
	0	250	500	1000	2000		
Yolk weight (g)	15.54	15.99	15.58	15.68	15.63	0.09	0.58
Alb weight (g)	35.28 ^a^	34.45 ^a^	36.25 ^a,b^	36.63 ^b^	37.13 ^b^	0.25	<0.01
Alb height (mm)	8.21	7.96	8.20	8.08	8.29	0.04	0.08
Haugh unit	90.74	88.69	89.74	88.79	90.02	0.27	0.08
Eggshell weight (g)	7.89	7.87	7.99	7.98	8.06	0.03	0.46
Shell thickness (mm)	0.41 ^a^	0.39 ^b^	0.39 ^b^	0.41 ^a^	0.41 ^a^	0.00	<0.01
Strength (Kgf)	3.95	4.06	3.98	4.21	4.18	0.04	0.17

^a,b^ Mean values with different letters in the same row indicate significant differences. SEM, standard error of measurement; Alb weight, Albumin weight; Alb height, Albumin height.

**Table 6:** The effect of *Caesalpinia sappan* extract supplementation on blood biochemistry of laying hens in late phase of laying cycle.

Parameters	Sappan-Containing Feeds (mg/kg Diet)					Pooled SEM	*p*-Value
	0	250	500	1000	2000		
AST (U/L)	249.00	268.70	321.70	278.70	242.70	46.1	0.76
ALT (U/L)	0.93	0.93	0.93	0.93	0.93	0.03	1.00
ALP (U/L)	465.30	573.30	1095.00	712.00	539.00	142	0.06
Total protein (mg/dL)	5.47	5.80	5.77	5.63	5.80	0.41	0.97
Globulin (mg/dL)	3.37	3.60	3.90	3.40	3.77	0.37	0.82
Albumin (mg/dL)	2.10 ^a,b^	2.20 ^a,b^	1.87 ^a^	2.23 ^b^	2.03 ^a,b^	0.08	0.04
A/G ratio	0.63	0.62	0.50	0.67	0.55	0.05	0.15
Total bilirubin (mg/dL)	0.16	0.14	0.15	0.14	0.16	0.01	0.22
Direct bilirubin (mg/dL)	0.04	0.04	0.07	0.05	0.05	0.01	0.45
Indirect bilirubin (mg/dL)	0.12	0.10	0.09	0.09	0.11	0.02	0.60

^a,b^ Mean values with different letters in the same row indicate significant differences (*p* < 0.05). ALP, Alkaline phosphatase; ALT, Alanine transaminase; AST, Aspartate transaminase; A/G ratio, Albumin/Globulin ratio.

**Table 7:** The effect of *Caesalpinia sappan* extract supplementation on body weight and the organ weights (in grams) of laying hens in late phase of laying cycle.

Organs	Sappan-Containing Feeds (mg/kg Diet)					Pooled SEM	*p*-Value
	0	250	500	1000	2000		
Whole body	1692.00	1708.00	1672.00	1732.00	1670.00	62.4	0.89
Liver	28.83	24.86	28.06	24.90	25.46	1.59	0.10
Spleen	2.83	2.23	2.95	1.79	2.94	0.60	0.28
Heart	8.26	7.94	8.42	8.94	8.74	1.14	0.27
Gizzard	27.36	30.41	31.02	30.35	33.82	1.97	0.18
Proventriculus	7.85	8.19	8.63	7.82	8.18	3.38	0.39
Intestine	83.18	83.26	80.66	87.14	84.57	5.54	0.95

**Table 8:** The effect of *Caesalpinia sappan* extract supplementation on intestinal morphology of laying hens in late phase of laying cycle.

Items	Sappan-Containing Feeds (mg/kg Diet)					Pooled SEM	*p*-Value
	0	250	500	1000	2000		
Duodenum							
VH	1436.00 ^a^	1697.00 ^a^	2168.00 ^b^	1601.00 ^a^	2567.00 ^c^	48.1	<0.01
VW	37.77 ^a^	60.28 ^a^	66.50 ^a,b^	99.48 ^b^	178.80 ^c^	5.93	<0.01
CD	295.20 ^a^	302.40 ^a,b^	325.90 ^a,b^	317.30 ^a,b^	372.80 ^b^	8.39	0.02
CA	2091.00 ^a^	3244.00 ^a,b^	3456.00 ^b,c^	2699.00 ^a,b^	4635.00 ^c^	158	<0.01
Jejunum							
VH	1220.00 ^a^	1351.00 ^a^	1427.00 ^a,b^	1575.00 ^b^	2106.00 ^c^	36.89	<0.01
VW	102.30 ^a^	180.80 ^c^	105.10 ^a^	121.70 ^a,b^	165.90 ^b,c^	5.83	<0.01
CD	245.90 ^a^	247.20 ^a^	270.90 ^a,b^	256.90 ^a^	330.10 ^b^	8.00	<0.01
CA	2308.00 ^a^	2326.00 ^a^	2431.00 ^a^	2757.00 ^a^	3950.00 ^b^	126	<0.01
Ileum							
VH	1014.00 ^a^	1194.00 ^a,b^	1257.00 ^b^	1081.00 ^a,b^	1196.00 ^a,b^	23.25	<0.01
VW	98.26 ^a^	158.80 ^b^	143.70 ^b^	131.70 ^b^	141.80 ^b^	4.06	<0.01
CD	237.90 ^a^	210.30 ^a^	245.70 ^a^	187.00 ^b^	206.10 ^a,b^	6.02	0.01
CA	1741.00	1552.00	2367.00	2168.00	2378.00	121	0.10

^a,b,c^ Within a row, mean without a common superscript differ (*p* < 0.05). VH, villus height; VW, villus width; CD, crypt depth; CA, crypt area.

## Data Availability

The data presented in this study are available on request from the corresponding author. The data are not publicly available due to privacy restrictions.
